# The Cambridge Face Tracker: Accurate, Low Cost Measurement of Head Posture Using Computer Vision and Face Recognition Software

**DOI:** 10.1167/tvst.5.5.8

**Published:** 2016-09-30

**Authors:** Peter B. M. Thomas, Tadas Baltrušaitis, Peter Robinson, Anthony J. Vivian

**Affiliations:** 1 Department of Ophthalmology, Addenbrooke's Hospital, Cambridge, UK; 2 Language Technologies Institute, Carnegie Mellon University, Pittsburgh, USA; 3 Computer Laboratory, University of Cambridge, Cambridge, UK

**Keywords:** head posture, computer vision, strabismus

## Abstract

**Purpose:**

We validate a video-based method of head posture measurement.

**Methods:**

The Cambridge Face Tracker uses neural networks (constrained local neural fields) to recognize facial features in video. The relative position of these facial features is used to calculate head posture. First, we assess the accuracy of this approach against videos in three research databases where each frame is tagged with a precisely measured head posture. Second, we compare our method to a commercially available mechanical device, the Cervical Range of Motion device: four subjects each adopted 43 distinct head postures that were measured using both methods.

**Results:**

The Cambridge Face Tracker achieved confident facial recognition in 92% of the approximately 38,000 frames of video from the three databases. The respective mean error in absolute head posture was 3.34°, 3.86°, and 2.81°, with a median error of 1.97°, 2.16°, and 1.96°. The accuracy decreased with more extreme head posture. Comparing The Cambridge Face Tracker to the Cervical Range of Motion Device gave correlation coefficients of 0.99 (*P* < 0.0001), 0.96 (*P* < 0.0001), and 0.99 (*P* < 0.0001) for yaw, pitch, and roll, respectively.

**Conclusions:**

The Cambridge Face Tracker performs well under real-world conditions and within the range of normally-encountered head posture. It allows useful quantification of head posture in real time or from precaptured video. Its performance is similar to that of a clinically validated mechanical device. It has significant advantages over other approaches in that subjects do not need to wear any apparatus, and it requires only low cost, easy-to-setup consumer electronics.

**Translational Relevance:**

Noncontact assessment of head posture allows more complete clinical assessment of patients, and could benefit surgical planning in future.

## Introduction

Ophthalmologic, neurologic, and orthopedic diseases can cause abnormal head posture^1^ (AHP). In ophthalmology, the measurement of AHP is particularly important in the assessment of strabismus, nystagmus, and ptosis. The head may be positioned abnormally in three dimensions. The head may be tilted towards one shoulder (head tilt or roll), the face may be turned to the right or left (face turn or yaw), and the chin may be tipped up or down (chin up/down or pitch). The classic example is seen in fourth (trochlear) nerve palsy: to maintain single vision, the patient must tilt his head away from the affected side, turn his face towards the affected side, and depress his chin. Over time this head posture can lead to self-consciousness and secondary muscular and skeletal problems in the neck.

The importance of measuring AHP is to determine the extent of the abnormality and to monitor changes in the patient's condition. Although we often comment on these variables, we do not always quantify them. This unsatisfactory situation arises because the existing methods of quantifying AHP are expensive, inconvenient, difficult to use, or not suitable for use on patients with poor compliance (especially children).

Traditional approaches to measuring AHP have used head-mounted equipment, or visual recording with a goniometer. The most popular contemporary example is the Cervical Range of Motion device (CROM; Performance Attainment Associates, St. Paul, MN). This has been modified and validated for use in ophthalmology,^2^ and it serves as the de facto gold standard against which novel methods have been tested.^3,4^ It relies on two inclinometers to measure chin up/down position and head tilt (mounted near the temple and forehead, respectively), while a magnetic compass (mounted over the vertex of the skull) measures rotation relative to a shoulder-mounted magnet (with the patient's shoulders aligned along magnetic North to avoid error). Although it is a sensible approach, there is the risk of variability in how the equipment is mounted, it is not suitable for young children, and there is the possibility that its presence will alter the head posture.

Recently, a number of publications have reported the use of electronic AHP measuring devices. Head-mounted motion trackers have been used to achieve head posture measurement,^5,6^ and Nintendo Wiimotes (Nintendo Co., Kyoto, Japan) have been used to construct an infrared head tracker.^4^ The former approach has the drawback of considerable expense, while the latter requires significant construction and calibration. Furthermore, both require head-mounted equipment, which risks altering head posture and restricts use to adults and compliant children.

A more promising approach involves the Microsoft Kinect (Microsoft Corp., Bellevue, WA) and Microsoft's Face-Tracking Software Development Kit to estimate head posture.^3^ This has the important advantage of freedom from head-mounted equipment. However, it is reliant on specialist equipment and software, which are liable to discontinuation, and would require significant programming skills to replicate.

We proposed the Cambridge Face Tracker as an ideal solution for head posture measurement. Using a standard webcam to capture live video of patients, a standard Windows-based PC analyzes head posture (roll, pitch, and yaw) in real time using our neural network–based software. The software also can analyze photos and videos that have been recorded previously on other devices.

We present two experiments: the first characterizes the accuracy of our technique against high precision head trackers and publically available head posture databases; the second compares the Cambridge Face Tracker with the CROM device (which has been used as comparator in several ophthalmic publications).^3,4^

## Methods

### The Cambridge Face Tracker

The Cambridge Face Tracker allows face detection using computer vision^7^ and runs on a standard Windows-based PC. It processes live video from any standard USB webcam (we used the Logitech C920HD, which has Carl Zeiss Meditec [Jena, Germany] optics in the present study) to detect faces and calculate their spatial position and orientation. This was achieved through a machine learning technique based on artificial neural networks called “Constrained Local Neural Fields.”^8^

The image first is scanned to find a face using standard Histogram of Oriented Gradient and Support Vector Machine–based face detection technique.^9^ The detected face area then is analyzed in more detail to find 68 facial locations (along the jawline, lid margins, lips, ridge of the nose, and eyebrows). The search is constrained by a simplified model of the face. Once the best fitting solution is determined, the orientation of the simplified model of the face is calculated yielding values for roll (head tilt), pitch (chin up/down), and yaw (face turn). The three-dimensional spatial relationship of the subject relative to the camera also is calculated in millimeters by assuming a standard interpupillary distance of 65 mm. A confidence value is calculated, which reflects the goodness of fit to the facial model, and, therefore, the likelihood that the calculated orientation is accurate: this will be negatively affected by factors, like rapid head movement, partial facial obscuration, and extreme abnormal head posture.

**Figure 1:**
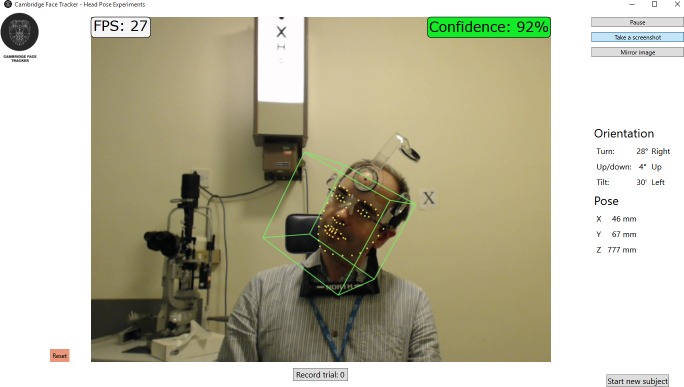
A screenshot of the Cambridge Face Tracker operating in a clinical setting. The subject is wearing the CROM device, but this does not interfere significantly with the performance of the Cambridge Face Tracker. The screenshot was taken while the software was running at 27 recordings per second (*top left*). The orientation of the captured head pose is displayed on the far right. The confidence value (*top right*) describes the goodness-of-fit of the facial model to the captured head. The value of 92% in this image represents a confident fit.

Setting up the webcam is straightforward. It is positioned at head height and leveled with a spirit level. The patient should be 1 to 1.5 m from the camera with their shoulders flat against the back of an examination chair. The chair should be oriented face on to the camera. In this way, the head posture is measured relative to the shoulders and to the visual axis of the eye. Fixation targets can be presented to determine whether the head posture varies depending on the task. A pause function is provided in the software to capture head posture when the subject cannot remain still for long. There also is a function that allows images and videos captured using standard cameras to be loaded into the software for retrospective analysis. Figure 1 shows a screenshot of the software in action.

#### Study 1: Validation of the Cambridge Face Tracker

To assess the accuracy of the Cambridge Face Tracker, we evaluated its performance on three publically available datasets (the ICT-3DHP, BIWI, and Boston University datasets). These datasets comprise videos of subjects in office environments. Each frame in these videos is annotated with the instantaneous head posture, which was measured at the time of original capture using one of several high accuracy techniques. In each dataset, subjects move their heads rapidly in real world conditions with cluttered backgrounds and uneven illumination.

The videos in the ICT-3DHP dataset (Institute for Creative Technologies, University of Southern California, Los Angeles, CA)^10^ were captured using a Microsoft Kinect sensor. The dataset contains 10 videos of approximately 1400 frames each. Each video contains footage of a subject moving their head on instruction. We used only the standard color videos from this dataset (it also contains depth videos). The true head posture in this dataset was measured with a Polhemus Fastrak magnetic position tracker (Polhemus, Colchester, VT). This tracks the position of sensors (mounted on a baseball cap) in a magnetic field generated by a source unit, and is accurate to orientation changes of 0.12° (root mean squared error) at a distance of 1.2 m from the transmitter. Each frame is annotated with the measured head posture.

The BIWI dataset^11^ contains over 15,000 frames of 20 people (6 males and 14 females) recorded with a Microsoft Kinect sensor. The annotated head posture in these videos was derived using person-specific face range scanning. Again, we used only the color video output of the Kinect in our analysis. This dataset contains dropped frames, which make the task of facial tracking more difficult, and so serves as a useful stress test.

The Boston University head pose dataset^12^ contains 45 videos of 5 subjects with 200 frames each. The annotated head posture in these videos was derived using a Flock of Birds Tracker (Ascension Technology Corporation, Shelburn, VT), similar to that used in the ICT-3DHP dataset.

To assess the accuracy of the Cambridge Face Tracker, our software was set up to analyze the prerecorded videos in the three datasets rather than a live video feed. For each frame, the estimated head posture from the Cambridge Face Tracker (roll, pitch, and yaw) was compared to the gold standard measure of head posture provided with each video. We calculated the overall error of our method; the error in roll, pitch, and yaw individually; and the error when only high confidence frames were allowed.

#### Study 2: Comparison to the CROM Device

We sought to determine whether the output from the Cambridge Face Tracker agrees with the head posture measured by the CROM device. Four healthy volunteers were seated on a standard examination chair 1.2 m in front of a Logitech C920HD webcam (Logitech, Romanel-sur-Morges, Switzerland). The height of the chair was adjusted to achieve approximate centration of the face in the frame. Figure 1 shows the output of the Cambridge Face Tracker in this environment: we intentionally placed standard ophthalmic instrumentation in the background (for example, a Snellen chart and a slit-lamp) to determine performance in a realistic clinic environment. The CROM device was fitted according to the manufacturer's instructions (it also can be seen in Figure 1).

To generate a series of head postures distinct in yaw (face turn) and pitch (chin up/down), subjects were instructed to “look with their heads and not their eyes” at a series of targets on the wall behind the webcam in a random order. The targets were placed at 5° increments of viewing angles from null (the position of the webcam) to eccentricities of ±40° in the pure horizontal (yaw) and pure vertical (pitch) meridians. To generate roll postures (head tilt), we instructed subjects to match their head tilt to a line oriented from −40° to +40° to the horizontal in 10° increments. Roll, pitch, and yaw were assessed independently. For each head posture we recorded the relevant value for roll, pitch, or yaw from the CROM device and the Cambridge Face Tracker. We also noted whether tracking had been lost in the Cambridge Face Tracker. This protocol does not force the subject to adopt a specific head posture, but serves simply to generate 43 distinct head postures (per subject). We measured 172 head postures in total across 4 subjects. For each of these poses, we compared the estimates of head posture from the Cambridge Face Tracker and the CROM device.

The experiments followed the tenets of the Declaration of Helsinki, informed consent was obtained from the subjects, and institutional ethical approval was obtained.

## Results

### Validity of the Cambridge Face Tracker

Summary data for the accuracy of the Cambridge Face Tracker in measuring head posture are shown in Table 1 for all frames in all databases, and also only those frames where head posture could be determined with high confidence (92% of all frames across the three datasets). The median error is presented in addition to the mean error as it is less affected by frames where head motion is rapid, head posture is extreme, or the face is partially obscured by hair falling in front of the face. These frames contribute the highest errors to the analysis, and are unlikely to be used in the clinical setting.

**Table.**
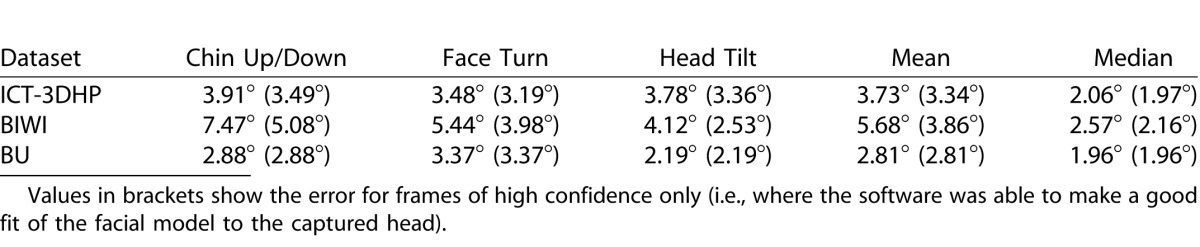
Mean Absolute Error in Degrees for Roll, Pitch and Yaw Independently and Combined

Subanalysis of the frames in all three datasets shows that the error increases with more extreme head posture. To achieve less than 5° mean absolute error (combined roll, pitch, and yaw) the operational range is ±30° head turn (yaw), ±20° chin up or down (pitch), and ±50° head tilt (roll).

**Figure 2:**
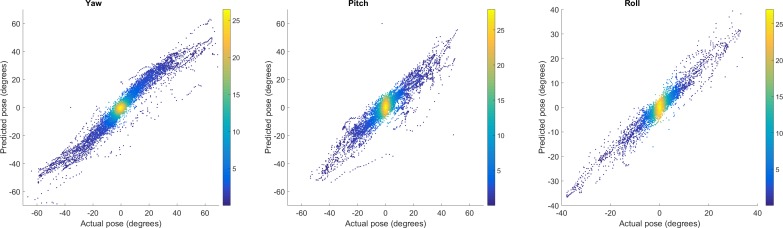
Comparison of the head pose predicted by the Cambridge Face Tracker (predicted pose) to that measured during original video capture (actual pose) for yaw, pitch, and roll (*top*). Due to the very high number of measurements (in excess of 30,000 frames were analyzed), the data are presented as a heat map.

To aid comparison with other published techniques, we also performed subanalysis on those frames where head posture deviated from the primary position predominantly only in roll, pitch, or yaw: respectively, the mean errors were 1.92°, 2.82°, and 2.14° with median errors 1.27°, 1.55°, and 1.35°. Figure 2 presents the estimated head posture against the actual head posture for roll, pitch, and yaw individually.

To give an indirect estimate of reproducibility, we identified subsets of frames where the same head pose was adopted by different subjects or at different times. Three subsets were extracted where the head pose deviated in only roll, pitch, or yaw from the straight ahead position. Owing to the paucity of discrete observations of some head poses, the dataset (for roll especially) was limited. For pitch ranging from −30° to +35° in 5° increments we identified 8 discrete measurements of each pose with an intraclass correlation coefficient (ICC) of 0.99 (average measures, single measure = 0.9). For yaw ranging from −35° to +35° we identified 11 discrete measures of each pose with an ICC of 0.99 (average measures, single measure = 0.96). For roll ranging from −15° to +15° we identified 4 discrete measures of each pose with an ICC of 0.98 (average measures, single measure = 0.92).

### Comparison to the CROM Device

**Figure 3:**
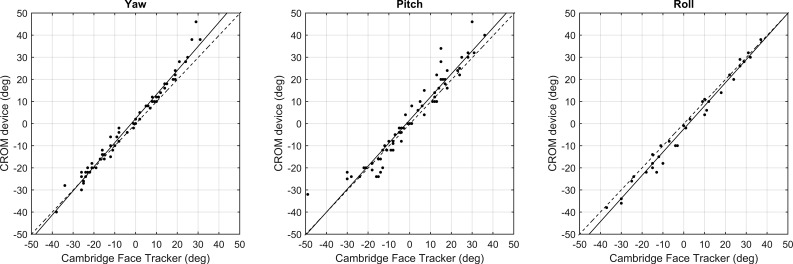
Comparison of the Cambridge Face Tracker and CROM device across 172 unique head postures: 68 postures varied in yaw (*left*), 68 in pitch (*middle*), and 36 in roll (*right*). The *dashed line* represents a theoretical line of perfect agreement (i.e., gradient of 1 and passing through the origin). *Solid lines* show linear regression between the two measures. These yield gradients of 1.09, 1.04, and 1.05, respectively, with *R*^2^ values of 0.98, 0.93, and 0.98, ordinate intersects of 2.4°, 1.61°, and 2.45°, and correlation coefficients of 0.99, 0.96, and 0.99 (yaw, pitch, and roll).

**Figure 4:**
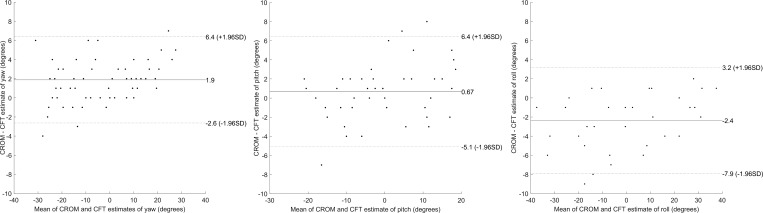
Bland-Altman plots of yaw, pitch, and roll (*left* to *right*) comparing the CROM device with the Cambridge Face Tracker (CFT). Only points within the accurate operational range (defined from Study 1) of ±30° yaw, ±20° pitch, and ±50° roll. This yields 65 data points for yaw, 45 for pitch, and 36 for roll.

Figure 3 compares the head posture measured by the Cambridge Face Tracker to that measured by the CROM device for roll (36 observations), pitch (68 observations), and yaw (68 observations). Linear regression analysis shows excellent agreement between the measures in yaw, pitch, and roll with *R*^2^ values of 0.98, 0.93, and 0.98, respectively. Correlation coefficients for yaw, pitch, and roll were 0.99 (*P* < 0.0001), 0.96 (*P* < 0.0001), and 0.99 (*P* < 0.0001). Bland-Altman analysis is shown in Figure 4 for those head poses that fall within the accurate operational range of the Cambridge Face Tracker (derived in Study 1).

## Discussion

Quantifying head posture is difficult in the clinical environment and so it is not often measured. This limits the assessment and monitoring of a number of conditions and prevents clinicians forming accurate management strategies. The ideal method for measuring abnormal head posture would be accurate and easy to use, and deploy noncontact, inexpensive equipment. It should be suitable for use in patients of all ages and give measurements in real time so that task-related behavior can be assessed (patients tend to adopt the largest head posture when performing a high spatial frequency task). The Cambridge Face Tracker has been designed to conform to these ideals.

In this study, we reported that The Cambridge Face Tracker showed good agreement with research-standard head posture measurements, though these research-standard methodologies have an accuracy far beyond that achievable with current clinical techniques. However, they are not easily transferrable to clinical practice: they require considerable expertise to set up, a dedicated space in which to be used, and are prohibitively expensive. The databases of head posture videos we used to validate The Cambridge Face Tracker are designed to be challenging, and contain dropped frames, cluttered backgrounds, uneven lighting, rapidly and unpredictably moving heads, and occasional obscuration of parts of the face. We expect the performance of the Cambridge Face Tracker on these databases of videos represents an underestimate of the accuracy that is obtainable in the normal clinical setting where the parameters will be more forgiving. It also should be noted that the videos used in validation are exclusively of adults.

The absolute errors achieved by the Cambridge Face Tracker in the validation study compare well to other published methods for clinical measures of AHP.^3,4^ Mean error when considering only high confidence frames (confidence level is displayed in real time by our software) is less than 4° across all datasets and degrees of freedom of motion. Median error, a more representative measure of clinic performance, since it reduces the impact of low accuracy frames which would be ignored in clinic, is approximately 2° across all datasets. This level of characterization of AHP would be clinically useful. Accuracy of the Cambridge Face Tracker does suffer as head posture becomes extreme. However, the low-error operational range of ±30° yaw, ±20° pitch, and ±50° head roll should cover the vast majority of head postures presenting to clinic. Future revisions might extend our operational range.

Our comparison study against the CROM device shows excellent agreement with the Cambridge Face Tracker. We believe the CROM device has significant disadvantages for the measurement of abnormal head posture that limit its accuracy. For example, the strap can be adjusted to vary the fit, and this will lead to error particularly in chin up/down measurement. Moreover, the gradations on the dials are marked only at 2° intervals, and it is not suitable for use on young children. However, since it has been clinically validated in ophthalmology, we believed it was important to show agreement with our technique.

Compared to other recent techniques, The Cambridge Face Tracker is easy and inexpensive to set up using hardware available in any home or office, and downloadable software. It does not require a dedicated clinic space and we have shown that it is sufficiently accurate to be clinically useful. This means that any clinic that treats patients with ocular motility conditions can measure abnormal head postures as part of the routine work-up. This will allow assessment of change in head posture (for instance in recovering cranial nerve paresis or pre- and postoperative change) with accurate and reproducible measurements. With future iterations of the software we hope to increase our accuracy further.

## References

[1] Nucci P, Kushner BJ, Serafino M, Orzalesi N. A multi-disciplinary study of the ocular, orthopedic, and neurologic causes of abnormal head postures in children. *Am J Ophthalmol*. 2005 ; 140: 65–68. 16038652 10.1016/j.ajo.2005.01.037

[2] Kushner BJ. The usefulness of the cervical range of motion device in the ocular motility examination. *Arch Ophthalmol*. 2000 ; 118: 946–050. 10900108

[3] Oh B-L, Kim J, Kim J, Hwang J-M, Lee J. Validity and reliability of head posture measurement using Microsoft Kinect. *Br J Ophthalmol*. 2014 ; 98: 1560–1564. 25271910 10.1136/bjophthalmol-2014-305095

[4] Kim J, Nam KW, Jang IG, Yang HK, Kim KG, Hwang JM,. Nintendo Wii remote controllers for head posture measurement: accuracy, validity and reliability of the infrared optical head tracker. *Invest* *Ophthalmol Vis Sci*. 2012; 53: 1388–1396. 10.1167/iovs.11-8329 22297495

[5] Hald ES, Hertle RW, Yang D. Application of a digital head-posture measuring system in children. *Am J Ophthalmol*. 2011 ; 151: 66–70. 21035783 10.1016/j.ajo.2010.07.013

[6] Hald ES, Hertle RW, Yang D. Development and validation of a digital head posture measuring system. *Am J Ophthalmol*. 2009 ; 147: 1092–1100. 19268892 10.1016/j.ajo.2008.12.026

[7] Baltrusaitis T, Robinson P, Morency L-P. Constrained local neural fields for robust facial landmark detection in the wild. In: *Proceedings of the IEEE* *International Conference on Computer Vision Workshops*. New York, NY: IEEE; 2013: 354–361.

[8] Baltrusaitis T, Robinson P, Morency L-P. Continuous conditional neural fields for structured regression. In: Fleet D, Pajdla T, Schiele B, Tuytelaars T eds. *Computer Vision –ECCV* 2014 New York: Springer; 2014: 593–608.

[9] King DE. Dlib-ml: A Machine Learning Toolkit. *J Machine Learn Res*. 2009; 10: 1755–1758.

[10] Baltrusaitis T, Robinson P, Morency L-P. 3D Constrained local model for rigid and non-rigid facial tracking. In: *IEEE Conference on Computer Vision and Pattern Recognition (CVPR)* . 2012.

[11] Fanelli G, Dantone M, Gall J, Fossati A, Van Gool L. Random forests for real time 3d face analysis. *Int J Comput Vis*. 2013 ; 101: 437–458.

[12] La Cascia M, Sclaroff S. Fast, reliable head tracking under varying illumination. *Comput. Vis. Pattern Recognition*, 1999 IEEE Comput. Soc. Conf. on. 1, 610 Vol. 1.

